# Cardiac Mesenchymal Stem Cell-like Cells Derived from a Young Patient with Bicuspid Aortic Valve Disease Have a Prematurely Aged Phenotype

**DOI:** 10.3390/biomedicines10123143

**Published:** 2022-12-06

**Authors:** Rachel A. Oldershaw, Gavin Richardson, Phillippa Carling, W. Andrew Owens, David J. Lundy, Annette Meeson

**Affiliations:** 1 Department of Musculoskeletal and Ageing Science, Institute of Life Course and Medical Sciences, Faculty of Health and Life Sciences, University of Liverpool, William Henry Duncan Building, 6 West Derby Street, Liverpool L7 8TX, UK; 2 Newcastle University Bioscience Institute, Newcastle University, International Centre for Life, Central Parkway, Newcastle upon Tyne NE1 3BZ, UK; 3 Department of Cardiothoracic Surgery, South Tees Hospitals NHS Foundation Trust, Middlesbrough TS4 3BW, UK; 4 Graduate Institute of Biomedical Materials and Tissue Engineering, Taipei Medical University, Taipei 110, Taiwan

**Keywords:** cardiac mesenchymal stem cell-like cells, bicuspid aortic valve disease, coronary artery disease, mesenchymal stem cells, ageing, senescence

## Abstract

There is significant interest in the role of stem cells in cardiac regeneration, and yet little is known about how cardiac disease progression affects native cardiac stem cells in the human heart. In this brief report, cardiac mesenchymal stem cell-like cells (CMSCLC) from the right atria of a 21-year-old female patient with a bicuspid aortic valve and aortic stenosis (referred to as biscuspid aortic valve disease BAVD-CMSCLC), were compared with those of a 78-year-old female patient undergoing coronary artery bypass surgery (referred to as coronary artery disease CAD-CMSCLC). Cells were analyzed for expression of MSC markers, ability to form CFU-Fs, metabolic activity, cell cycle kinetics, expression of NANOG and p16, and telomere length. The cardiac-derived cells expressed MSC markers and were able to form CFU-Fs, with higher rate of formation in CAD-CMSCLCs. BAVD-CMSCLCs did not display normal MSC morphology, had a much lower cell doubling rate, and were less metabolically active than CAD-CMSCLCs. Cell cycle analysis revealed a population of BAVD-CMSCLC in G2/M phase, whereas the bulk of CAD-CMSCLC were in the G0/G1 phase. BAVD-CMSCLC had lower expression of NANOG and shorter telomere lengths, but higher expression of p16 compared with the CAD-CMSCLC. In conclusion, BAVD-CMSCLC have a prematurely aged phenotype compared with CAD-CMSCLC, despite originating from a younger patient.

## 1. Introduction

A number of resident cardiac stem cell populations have been identified, several of which are present in the human heart [1,2,3,4,5]. However, clinical trials utilizing bone marrow or cardiac-derived stem cells have delivered only modestly successful results, with only a few patients showing meaningful improvements in cardiac function [6,7].

It is now accepted that most therapeutic benefits of MSC transplantation for cardiac repair are driven by paracrine secretions of numerous growth factors, cytokines and extracellular vesicles that aid in promoting cardiomyocyte survival, angiogenesis, and steering remodeling towards a less fibrotic phenotype [8]. Despite this, their mechanisms of action within the heart remain controversial and there has also been consideration of their possible role in tumor formation [9,10]. Further challenges are presented in the production of clinically relevant cell therapy products, including the influence of in vitro ageing during culture expansion [11], and limited retention and survival of donor cells at the target site, which requires combination of cells with supporting biocompatible materials [12,13,14,15,16]. 

It is well-known that not all donor cells are created equal [17]. For example, a previous study has shown that myocardial infarction adversely influences the therapeutic potential of bone marrow-derived cells from the same donor [18]. Furthermore, the secretome varies dynamically according to phenotype of the originating cells. Extracellular vesicles derived from post-MI mouse hearts were shown to aggravate inflammation and worsened heart function in other animals [19]. Thus, the source of donor therapeutic cells might also impact on the therapeutic outcome. By their very nature, patients requiring cardiac surgical intervention are rarely healthy or absent of cardiovascular disease. Therefore, if cardiac-derived cells are to be used for therapy, better understanding of the effects of cardiac disease and/or aging on those cells is needed.

Coronary artery disease (CAD) is a common disease caused by the buildup of atheroma in the coronary arteries that causes narrowing of the arteries and reduced blood flow to the heart [20]. This atherosclerotic process is, however, not restricted to the coronary arteries or even to the heart and can affect a number of different organs in the body and can therefore be considered a systemic disease [21]. The loss and/or dysfunction of MSCs has been associated with many systemic diseases (reviewed in Vizoso et al., 2019 [22]), while circulating endothelial progenitor cells (CPCs) expressing osteocalcin have been shown to be present in higher numbers in patients with coronary atherosclerosis than those without [23]. These studies support a role for progenitors/stem cells as either contributing to, or as being targets of, disease progression in some systemic diseases, including CAD. Contrary to this, bicuspid aortic valve defects are a form of congenital heart defect, often associated with several other cardiac complications, the symptoms of which frequently become apparent with increasing age [24,25].

One cell fate known to impede stem cell function is cellular senescence, controlled by the p16-pRb and p53-p21 pathways and defined as a cell cycle arrest, alterations in gene and protein expression and the production of the senescence-associated secretory phenotype (SASP) [26], a cocktail of pro-inflammatory cytokines, chemokines, matrix proteases and growth factors, which in the heart can impact tissue function, attenuate regeneration, induce fibrosis, extracellular matrix degeneration and drive inflammation [27]. Cardiac regenerative potential declines with age [28] and populations of human cardiac progenitor cells (CPCs) accumulate the senescence phenotype with age, express p16 and are unable to replicate, differentiate, regenerate or restore cardiac function following transplantation into the infarcted heart [29]. Our recent studies demonstrate that in the heart, cardiomyocyte, fibroblast and endothelial senescence can be induced not only by ageing but also as a result of cellular stress and disease [30,31]. The effect of disease on progenitor cells independent of ageing has not yet been investigated.

We have previously reported on the identification of human cardiac mesenchymal stem cell-like cells (CMSCLC), which have stem cell-like characteristics and an immunophenotype typical of MSC. These cells were capable of low levels of adipogenic differentiation but failed to differentiate into osteoblasts or chondrocytes. However, these CMSCLC did, under cardiac differentiation conditions, have the phenotype of both mature and immature cardiac cells, expressing troponin C and Nkx2.5, respectively [5]. CMSCLC express many of the bioactive molecules that make up the cardio-beneficial paracrine secretome, including, interleukin-10 (IL10), fibroblast growth factor-2 (FGF2), vascular endothelial growth factor (VEGF), transforming growth factor (TGF) and hepatocyte growth factor (HGF) [5], and their potential for therapeutic application demonstrated in vivo by improved cell retention, survival, extracellular vesicle production, and promotion of functional cardiac repair when encapsulated and delivered to a murine model of myocardial infarction ischemic injury [32].

Clinical presentation of a 21-year-old female patient with relatively rare bicuspid aortic valve disease (BAVD) provided an opportunity to present a case-based study that contributes to advancing the understanding of the impact of both age and different cardiomyopathies on the health of CMSCLC, and thus the potential implications for application of cardiac-derived stem cell-based therapies in clinic. We have isolated and compared CMSCLC from the atrial appendage of both the BAVD patient and from a 78-year-old female patient with coronary artery disease (CAD). We provide evidence that CMSCLC from the 21-year-old BAVD patient have a prematurely aged phenotype when compared with CMSCLC from the 78-year-old CAD patient. This suggests that premature aging of resident cardiac stem cells may contribute to additional cardiac complications observed in some patients with BAVD. 

## 2. Materials and Methods

### 2.1. Cardiac MSC Isolation and Derivation

All cardiac tissue samples used in this study were collected from consenting patients undergoing cardiac surgery under REC number UKCRN ID: 20120092. Right atrial appendage CMSCLC were derived and cultured as described previously [5]. Assessment of colony-forming unit-fibroblast (CFU-F) formation was performed as described previously [33].

### 2.2. Immunophenotyping of Cardiac MSC Populations

Immunophenotyping of CMSCLC for expression of cell surface antigens, including CD44, CD73, CD90, CD105, CD106, CD146, CD166, CD19 and CD45 was performed and analyzed as described previously with all primary antibodies purchased from R&D Biosystems, Abingdon, UK [5]. 

### 2.3. Measurement of Metabolic Activity

The metabolic activity of CMSCLC as an indicator of cell health was measured by intracellular reduction of resazurin (IUPAC: 7-hydroxy-10-oxidophenoxazin-10-ium-3-one) to resorufin (absorbance = A_570_) using alamarBlue^®^ metabolic assay (Thermo Fisher Scientific, Paisley, UK) as previously described [33,34,35]. For normalization of cell numbers DNA was quantified using Quant-iT™ PicoGreen^®^ dsDNA Kit (Thermo Fisher Scientific) as previously described [33].

### 2.4. Measurement of Telomere Length

Measurement of telomere length was performed at passage 5 of culture using the TeloTAGGG Telomere Length Assay (Roche, St Albans, UK) according to manufacturer’s instructions. Genomic (g)DNA (5 μg) was purified using the QIAamp DNA mini kit (Qiagen, Manchester, UK) and digested in parallel with 1.5 μg positive control DNA with 20 U/μL each of restriction endonucleases Hinf I and Rsa I. Telomere fragments were resolved by 0.8% (wt/vol) agarose gel electrophoresis, blotted by capillary action onto nylon membrane and hybridized with a digoxigenin (DIG)-labelled telomere-specific hybridization probe. Telomere fragments were visualized by chemiluminescence following incubation with 75 mU/mL of anti-DIG antibody conjugated with alkaline phosphatase and CDP-Star chemiluminescence substrate. 

### 2.5. Immunocytochemistry

Cells were cultured on chamberslides and fixed in 4% (wt/vol) paraformaldehyde for 5 min and labelled with primary antibody specific to Nanog (dilution 1:200, ab62734, Abcam, Cambridge, UK) or p16 (dilution 1:50, J0411, Santa Cruz, Heidelberg, UK). An Alexa-488 conjugated secondary antibody (dilution 1:1000, Molecular Probes; Thermo Fisher Scientific) was used to detect primary labelling. Nuclei were labelled with 0.2 mg/mL 4′,6-diamidino-2-phenylindole (DAPI, Sigma, Poole, UK). Analysis was performed using an Axioimager M1 fluorescence microscope (Carl Zeiss) running OpenLab software (Improvision). The specificity of all secondary antibodies and the absence of autofluorescence were tested by omitting primary control. To allow comparison of protein expression level in the different patient samples the same exposure time was used for each sample.

### 2.6. Cell Cycle Analysis

Cell cycle analysis was carried out using the CyStain DNA 2 step kit (Partec, Wymbush, UK) CMSCs at 90% confluence were resuspended in 100 μL extraction buffer and incubated at room temperature for 15 min. Cells were labelled with the addition of 500 μL staining solution (containing DAPI) and incubated overnight at 4 °C. Flow cytometry was performed on FACS Canto (BD) using 405 450/50 filters.

## 3. Results

### 3.1. Colony-Forming Unit Fibroblast Potential, Cell Derivation Rate and Morphological Analysis of CMSCCLC

**Figure 1:**
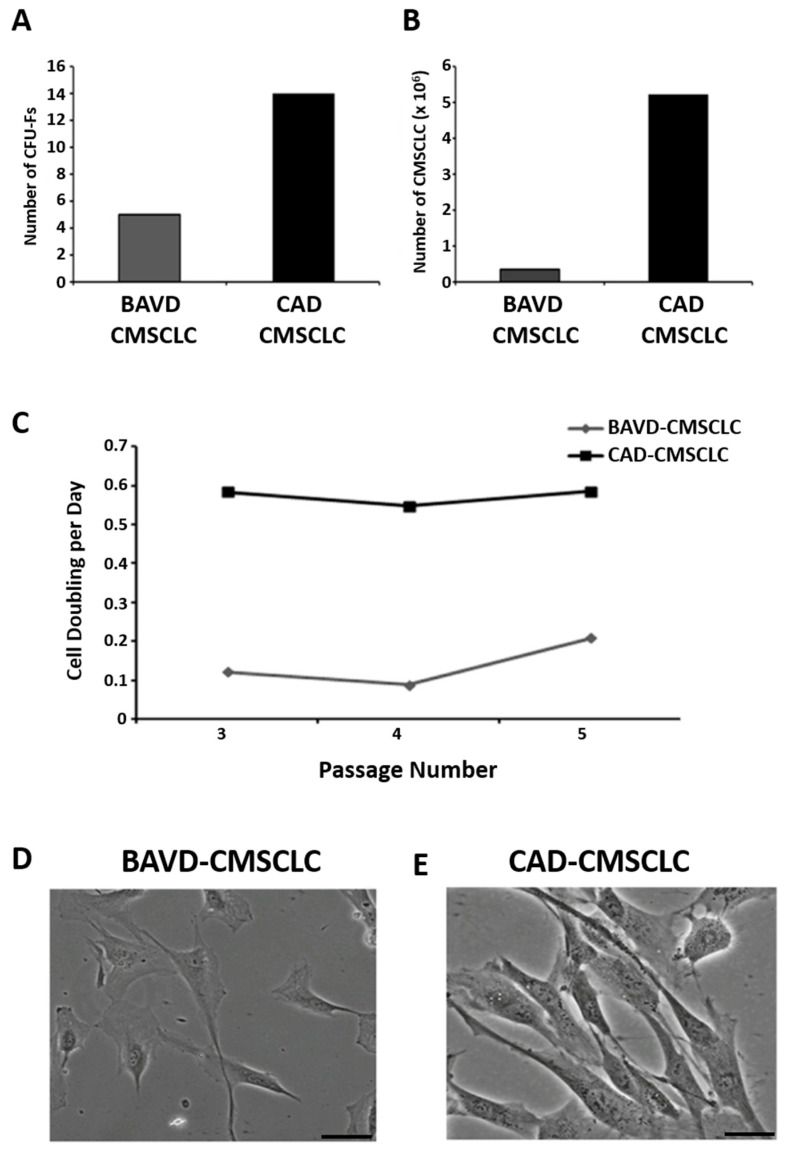
Derivation and characterization of CMSCLC from BAVD and CAD tissue. (**A**) Derivation of CMSCLC from BAVD and CAD cardiac tissue was quantified by counting the formation of established CFU-Fs. (**B**) Total number of CMSCLC counted from established CFU-Fs. (**C**) Quantification of cell doubling per day for BAVD-CMSCLC and CAD-CMSCLC over passages 3–5 of in vitro culture. (**D**,**E**) Brightfield images of BAVDMSCs and CADMSCs. Scale bar = 50 μm.

Comparative analysis of CMSCLC in vitro derivation was made between BAVD and CAD tissues. The ability of CMSCLC to form colony-forming unit fibroblasts (CFU-Fs) was examined with BAVD-CMSCLC producing a total of 5 CFU-Fs, fewer than the CAD-CMSCLC, which formed 14 CFU-Fs (Figure 1A). The number of cells that could be derived from these CFU-Fs was determined at passage 1. BAVD-CMSCLC CFU-Fs gave rise to 3.34 × 10^5^ cells and CAD-CMSCLC CFU-Fs gave rise to 5.5 × 10^6^ cells (Figure 1B). To allow time for cells to adapt from an in vivo to an in vitro environment, cell doublings were assessed at passages 3–5 and showed that BAVD-CMSCLC had a much lower cell doubling rate than CAD-CMSCLC (Figure 1C). The morphology of BAVD- and CAD-CMSCLC was examined at passage 5. BAVD-CMSCLC appeared ‘prematurely aged’, being flatter and more irregular in shape with large cytoplasmic volume in comparison to CAD-CMSCLC, which had a more typical fibroblastic MSC morphology, having a small cell body with long cellular protrusions (Figure 1D,E). 

### 3.2. CMSCLC Immunophenotyping

**Figure 2:**
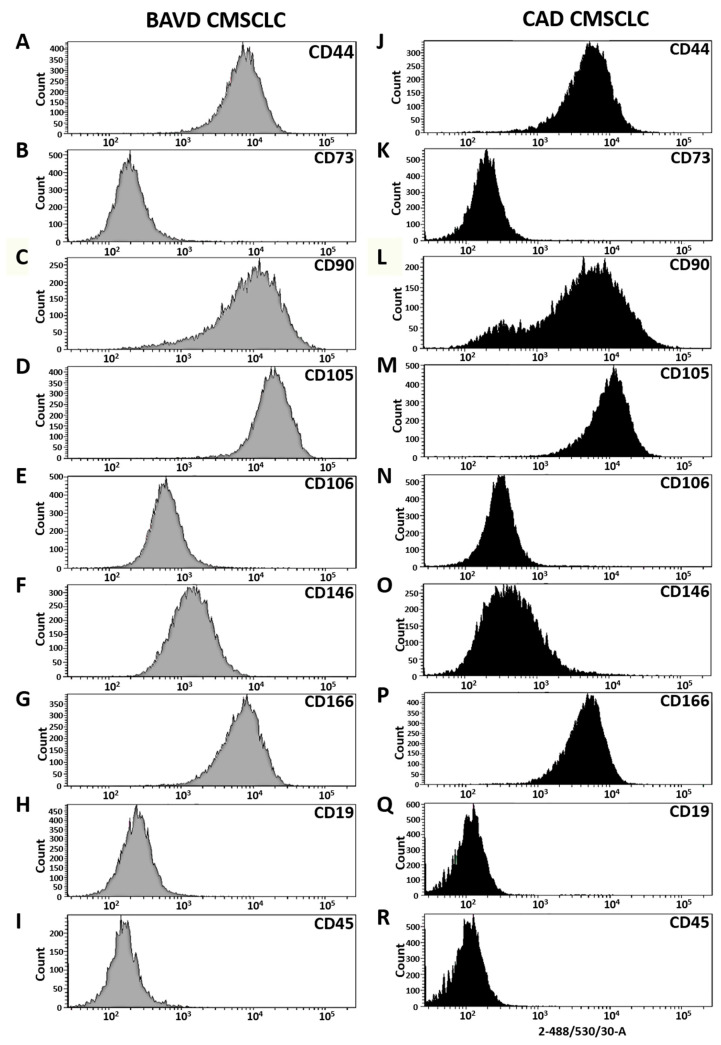
Immunophenotyping of MSC-associated cell surface antigens. Results for CMSCLCs immunolabelled with antibodies to human CD cell surface antigens. BAVD-CMSCLC express a number of markers of MSCs (**A**–**G**) but are negative for CD19 (**H**) and CD45 (**I**). CAD-CMSCLC also express the expected MSC markers (**J**–**P**) but are also negative for CD19 (**Q**) and CD45 (**R**).

The immunophenotype of CMSCLC derived from BAVD and CAD tissues was determined by profiling a panel of cell surface antigens established as being positively or negatively expressed by MSC. CMSCLC were immunolabelled using antibodies to CD44, CD73, CD90, CD105, CD106, CD146, CD166, CD45 and CD19 and used in combination with flow cytometry [36]. Analysis of the data generated revealed that both cell populations expressed markers normally expressed by MSCs and CMSCLCs, and were negative for expression of CD45 and CD19 (Figure 2). 

### 3.3. Expression of NANOG and p16 in CMSCLC

**Figure 3:**
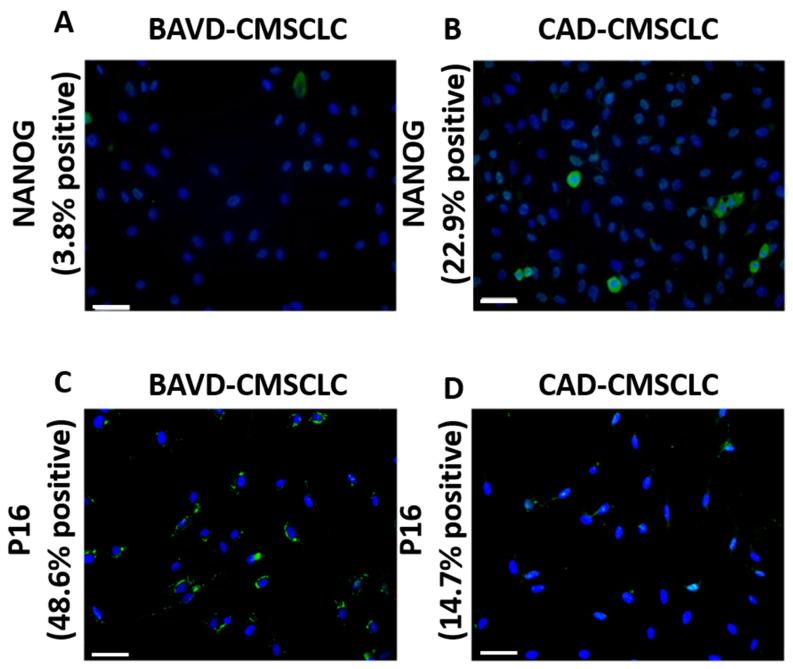
Immunolabelling of CMSCLC for NANOG and p16. (**A**,**B**) ICC analysis for NANOG expression in BAVD-CMSCLC and CAD-CMSCLC. NANOG (Green), all nuclei labelled with Dapi (Blue). (**C**,**D**) ICC analysis for p16 expression in BAVD-CMSCLC and CAD-CMSCLC. p16 (Green), all nuclei labelled with DAPI (Blue). Scale bar = 50μm.

Evaluation of the CMSCLC stem cell phenotype was performed by immunolabelling BAVD- and CAD-CMSCLC with anti-human NANOG antibody at passage 5. Nuclear-specific expression of NANOG was observed in both BAVD-CMSCLC and CAD-CMSCLC, however expression of NANOG appeared to be downregulated in BAVD-CMSCLC, being expressed in 3.8% of the cell population compared to 22.9% of CAD-CMSCLC (Figure 3A,B). 

In response to the ‘prematurely aged’ cellular morphology observed in BAV-CMSCLC, cells were also immunolabelled for p16, a key regulator of cellular senescence, which is upregulated in cells causal to systemic ageing [37], including senescent MSC [38] and senescent cardiac cells that contribute to myocardial remodeling [31,39], and which in a recent systemic review was identified as a robust marker for the detection of senescence in human tissue samples [40]. BAVD-CMSCLC were shown to have higher expression of p16 (48.6% of culture) compared to CAD-CMSCLC (14.7% of culture) (Figure 3C,D). 

### 3.4. Evaluation of Cellular Ageing My Measurement of Telomere Length, Metabolic Activity and Cell Cycle Kinetics

**Figure 4:**
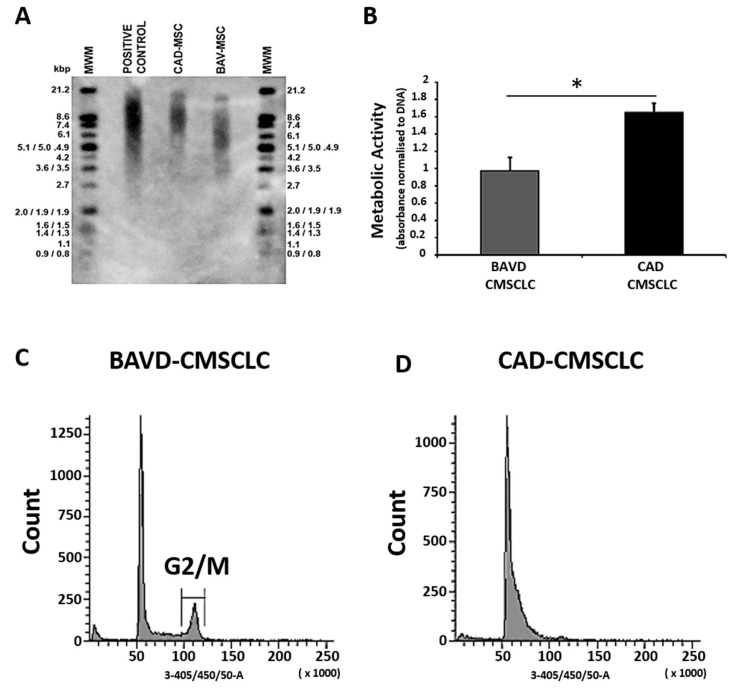
Evaluation of CMSCLC senescent phenotype (**A**) Measurement of CMSCLC telomere length. (**A**) TeloTAGGG Telomere Length Assay was used to measure telomere length of BAVD-CMSCLC and CAD-CMSCLC. Resolved telomere fragments from Hinf I/Rsa I-digested control gDNA, CAD-CMSCLC gDNA and BAV-CMSCLC gDNA were hybridized with DIG-labelled telomere-specific probe, incubated with anti-DIG antibody conjugated to alkaline phosphatase and visualized by chemiluminescence. Average telomere fragment lengths were calibrated against a DIG-labelled molecular weight marker (MWM). (**B**) Measurement of metabolic activity. Measurement of metabolic activity as an indicator of cell health was performed on CMSCLC using an alamarBlue^®^ assay, the fluorescence of the resulting culture media was normalized against total DNA content. * = *p* < 0.05 of *n* = 3 independent technical replicates, Mann–Whitney test. (**C**,**D**) Cell cycle analysis. Results of flow cytometry analysis for cell cycle of BAVD-CMSCLC, note the presence of a subpopulation of cells in the G2/M phase that is absent in the CAD-MSCs.

Further comparative investigation of cellular ageing in BAV-CMSCLC and CAD-CMSCLC was performed at passage 5. Analysis of telomere length demonstrated that BAVD-CMSCLC had shorter telomeres (5.3 kbp) than CAD-CMSCLC (8.3 kbp) (Figure 4A).

Metabolic activity was also measured using an alamarBlue^®^ assay normalized to total DNA and was lower in BAVD-CMSCLC compared to CAD-CMSCLC (Figure 4B). CMSCCL were also stained and analyzed for cell cycle progression, which showed a population of BAVD-CMSCLC in G2/M phase, whereas the bulk of CAD-CMSCLC were in the G0/G1 phase of the cell cycle (Figure 4C,D). 

## 4. Discussion

Bicuspid aortic valve defects are a common form of congenital heart defect, often associated with a number of other cardiac complications, the symptoms of which frequently become apparent with increasing age [24,25]. The causes of BAVD, however, remain unclear. We report for the first time on differences between CMSCLC isolated from of a young female patient with BAVD disease and an elderly female patient with CAD disease. We observed that both the CAD-CMSCLC and BAVD-CMSCLC expressed cell surface makers normally expressed by MSCs and lack expression of hematopoietic lineage makers. Moreover, the CMSCLCs adhere to plastic under standard MSC culture conditions [41,42]. However, morphologically, while the CAD-CMSCLC displayed an MSC-like morphology [41], the BAVD-CMSCLC did not; they displayed characteristics associated with aged MSCs [43].

One well-recognized stem cell characteristic is the ability to form colonies in culture; we observed that CAD-CMSCLC formed more CFU-Fs than the BAVD-CMSCLC. They also formed fewer CFU-Fs than for other patient-derived CMSCLC reported in our previous study [5]. Moreover, the number of cells that could be derived from the CAD-CFU-Fs was higher than those that could be derived from the BAVD-CFU-Fs. BAVD-CMSCLC also had a slower doubling rate and a reduced capacity for cell division compared with CAD-CMSCLC, both of which are characteristics of aging [43]. The BAVD-CMSCLC also had a shorter telomere length than the CAD-CMSCLC. The replicative potential of hematopoietic stem cells is related to telomere length and as such this provides an indication of stem cell function and there is increasing evidence that telomerase activity and telomere length are important for the function of bone marrow derived MSCs. Mouse MSCs that lack telomerase activity show an inability to differentiate into adipocytes or chondrocytes [44,45] while human MSCs, forced to overexpress telomerase, have increased proliferative potential [45]. It has been suggested that in human MSCs telomerase activity is required to bring about regenerative capacity and differentiation potential [46]. The shorter telomeres in the BAVD-CMSCLC may therefore be an indicator of the inferior quality of these cells compared with the CAD-CMSCLC and suggest that like bone marrow MSCs telomerase activity and telomere length is required to maintain the CMSCLC proliferative potential.

NANOG is associated with biological processes important for stem cell function including proliferation and differentiation potential [47,48,49]. In aging murine bone marrow MSCs, the over-expression of NANOG reversed ageing-associated loss of proliferation and myogenic potential [50]. We observed NANOG expression in both CMSCLC populations; however, there was decreased expression in terms of cell numbers in BAVD-CMSCLC. Furthermore, p16 an inhibitor of cell cycle progression most commonly associated with cell senescence including, tissue resident MSC and cardiomyocytes, was upregulated in BAVD-CMSCLC compared to CAD-CMSCLC [30,51,52]. While p16 is a robust marker of human senescence in vivo [40], the senescent phenotype can be heterogeneric and dependent on cell type and stimuli as such a deeper analysis of senescence-associated gene expression would provide insight into the signature of senescent CMSCLCs [53]. 

Cell cycle analysis revealed difference in cell cycle kinetics between the CAD and BAVD CMSCLC, with a subpopulation of BAVD cells being in the G2/M phase of the cell cycle which can be indicative of a stressed or senescent cell phenotype [50]. Recent studies have demonstrated that epigenetic biomarkers of ageing are prognostic of disease across multiple tissues including the cardiovascular system. [54,55,56]. While it is beyond the scope of the current study, a future investigation of epigenetic changes within CMSCLC populations could shed light on if epigenetic changes are associated with CMSCLC age and disease-related dysfunction.

Finally, we investigated the health of both populations of cells. Quantification of metabolic activity by alamarBlue^®^ assay is an established indicator of cell health whereby measurement of the reducing power of the intracellular environment includes contribution from mitochondrial and cytoplasmic reductases, and is therefore a function of both aerobic glycolysis and oxidative phosphorylation, respectively [34]. Our observation that the BAVD-CMSCLC were less metabolically active than the CAD-CMSCLC suggests that these cells have an impaired cellular metabolism, consistent with the established phenotype of senescent cells [34,57,58]. Metabolic balance is known to couple bioenergetic state with broader physiological pathways that regulate MSCs phenotype and function, including the metabolic switch of aerobic glycolysis to oxidative phosphorylation that drives differentiation [59]. Whilst aerobic glycolysis might be expected to be the predominant metabolic pathway in rapidly proliferating CMSCLC, the involvement of oxidative phosphorylation in the production of metabolite intermediates that form the precursors of biosynthetic pathways, as well as the metabolism of glutamine to glutathione for the regulation of REDOX signaling should also be considered [60,61,62]. Dysregulation of these integrated metabolic pathways is linked to elevated production of reactive oxygen species (ROS) and cellular oxidative stress that drives the senescent phenotype, and we propose this mechanism as one that warrants further interrogation in determining the health status of patient-derived CMSCLC [60,61].

The premature senescence evidenced in the BAVD-CMSCLCs is intriguing when considering that BAVD has no anatomical or embryological link with the right atrium (the aortic valve originates from neural crest and mutations tend not to affect right heart), whereas CAD associated with significant atherosclerosis is a systemic illness as opposed to a very organ-specific abnormality [63,64]. We suggest that in atherosclerotic cardiovascular disease, vascular remodeling, whilst being initiated by dysfunctional endothelial cells, is contributed to by stem cells originating from several sources. However, these cells have been shown to differentiate primarily to adipocytes, chondrocytes and osteocytes [65]. In our previously published study, CMSCLC from RAA of patients with CAD also had a very poor ability to undergo this tri-lineage differentiation [5], suggesting that CMSCLC from RAA are distinct from the stem cells contributing to cardiovascular disease. Resident cardiac stem cells known as CASCs, have also been isolated from atrial appendages based on aldehyde dehydrogenase activity and have been shown to be present in both human and pig heart. In the pig examination of different regions of the heart for the presence of CASCs revealed the highest numbers to be present in RAA and it is therefore a good source of stem cells that are relevant to cardiac-specific therapeutic application [66]. Interestingly, it has been proposed that individuals with BAV are predisposed to senescence [67]. Individuals with BAV display an increased prevalence of thoracic aortic aneurysm (TAA) and have a significantly increased risk of aortic dissection compared to those with a typical tricuspid valve (TAV), while senescence is associated with TAA in both TAV and BAV patients, only aortas of individuals with BAV contained senescence cells in the absence of TAA [68]. Observations that suggest BAV aortas have an increased predisposition to senescence even in the absence of symptomatic disease. Although the cause of this predisposition is unknown, however the genetics contributing to the disease may play a role, therefore it is possible that other cardiac resident cells including CMSCLCs share this susceptibility to senescence. 

The ultimate goal for stem cell research is cellular therapy; therefore, it is imperative that we fully understand how donor disease and genetics effects stem cell biology. Whilst our study is limited by access to cardiac tissue of one donor with relatively rare BAVD, our data suggests that genetic valvular disease can promote a senescence phenotype, and raises important questions regarding the appropriateness of using stem cells from diseased individuals, which may be required for autologous transplantation. Studies investigating age-related senescence have demonstrated that c-kit expressing cardiac progenitor cells have impaired stem cell function and express a proinflammatory SASP that promotes senescence in healthy CPC populations [29]. As such, cellular therapies that use cell populations, which include senescent cells may not only be less effective but may in fact be detrimental to both the transplanted population as a whole and the organ into which they are transplanted. Indeed, in animal models, transplantation of small numbers of senescent cells induces age-related disease, increases frailty, and increases mortality [6,7,8]. It is possible that in the future some of these challenges could be overcome isolated senescent CPCs can be “rejuvenated” via the treatment with senolytics which induce senescent but not proliferative cells to apoptosis [29].

## 5. Conclusions

In conclusion, our data suggest that the CMSCLC derived from the 21-year-old patient with BAVD disease were less metabolically active, less proliferative, had begun to show less ‘stemness’ characteristics and were prematurely aged compared with those of the 78-year-old patient with CAD. These findings have implications for the use of autologous cardiac derived stem cells for use as a therapeutic tool.

## Data Availability

Not applicable.
